# Basal Ganglia Neuromodulation Over Multiple Temporal and Structural Scales—Simulations of Direct Pathway MSNs Investigate the Fast Onset of Dopaminergic Effects and Predict the Role of Kv4.2

**DOI:** 10.3389/fncir.2018.00003

**Published:** 2018-02-06

**Authors:** Robert Lindroos, Matthijs C. Dorst, Kai Du, Marko Filipović, Daniel Keller, Maya Ketzef, Alexander K. Kozlov, Arvind Kumar, Mikael Lindahl, Anu G. Nair, Juan Pérez-Fernández, Sten Grillner, Gilad Silberberg, Jeanette Hellgren Kotaleski

**Affiliations:** ^1^Department of Neuroscience, Nobel Institute for Neurophysiology, Stockholm, Sweden; ^2^Bernstein Center Freiburg, University of Freiburg, Freiburg im Breisgau, Germany; ^3^Blue Brain Project, Ecole Polytechnique Fédérale de Lausanne, Geneva, Switzerland; ^4^Science for Life Laboratory, School of Electrical Engineering and Computer Science, KTH Royal Institute of Technology, Solna, Sweden; ^5^Department Computational Science and Technology, School of Electrical Engineering and Computer Science, KTH Royal Institute of Technology, Stockholm, Sweden

**Keywords:** striatum, medium spiny projection neurons, dopamine, simulations, Kv4.2, subcellular signaling, kinetic modeling

## Abstract

The basal ganglia are involved in the motivational and habitual control of motor and cognitive behaviors. Striatum, the largest basal ganglia input stage, integrates cortical and thalamic inputs in functionally segregated cortico-basal ganglia-thalamic loops, and in addition the basal ganglia output nuclei control targets in the brainstem. Striatal function depends on the balance between the direct pathway medium spiny neurons (D1-MSNs) that express D1 dopamine receptors and the indirect pathway MSNs that express D2 dopamine receptors. The striatal microstructure is also divided into striosomes and matrix compartments, based on the differential expression of several proteins. Dopaminergic afferents from the midbrain and local cholinergic interneurons play crucial roles for basal ganglia function, and striatal signaling via the striosomes in turn regulates the midbrain dopaminergic system directly and via the lateral habenula. Consequently, abnormal functions of the basal ganglia neuromodulatory system underlie many neurological and psychiatric disorders. Neuromodulation acts on multiple structural levels, ranging from the subcellular level to behavior, both in health and disease. For example, neuromodulation affects membrane excitability and controls synaptic plasticity and thus learning in the basal ganglia. However, it is not clear on what time scales these different effects are implemented. Phosphorylation of ion channels and the resulting membrane effects are typically studied over minutes while it has been shown that neuromodulation can affect behavior within a few hundred milliseconds. So how do these seemingly contradictory effects fit together? Here we first briefly review neuromodulation of the basal ganglia, with a focus on dopamine. We furthermore use biophysically detailed multi-compartmental models to integrate experimental data regarding dopaminergic effects on individual membrane conductances with the aim to explain the resulting cellular level dopaminergic effects. In particular we predict dopaminergic effects on Kv4.2 in D1-MSNs. Finally, we also explore dynamical aspects of the onset of neuromodulation effects in multi-scale computational models combining biochemical signaling cascades and multi-compartmental neuron models.

## Introduction

To survive and thrive it is crucial for all species to recognize and evaluate environmental cues, select actions and learn from previous behavioral outcomes (Redgrave et al., 1999). A brain area of specific importance in this task is the basal ganglia (BG), a set of interconnected nuclei, specialized in generating goal-directed and habitual motor- and cognitive behaviors.

The BG are centrally located in the forebrain and receive inputs from structures throughout the neuraxis, directly from different parts of the cortex and thalamus, or indirectly (via the thalamus) from many regions in the brainstem (Gerfen, 2004). The same brain structures (e.g., frontal cortex) that provide input to the BG also receive direct (via thalamus and brainstem structures) or indirect feedback from the BG output nuclei (Chevalier and Deniau, 1990). This architecture is often viewed as a series of partially segregated parallel projecting re-entrant loops involving both cortical and sub-cortical structures, controlling associated behaviors (Alexander et al., 1986; McHaffie et al., 2005). The moment to moment signaling through these BG loops are controlled by neuromodulation (see Hong, 2013), of which one of the most influential and studied neuromulatory system is dopamine (DA). Importantly, also learning/plasticity requires intact DA and acetylcholine (ACh) signaling (Knowlton et al., 1996; Matsumoto et al., 1999; Kitabatake et al., 2003).

**Figure 1:**
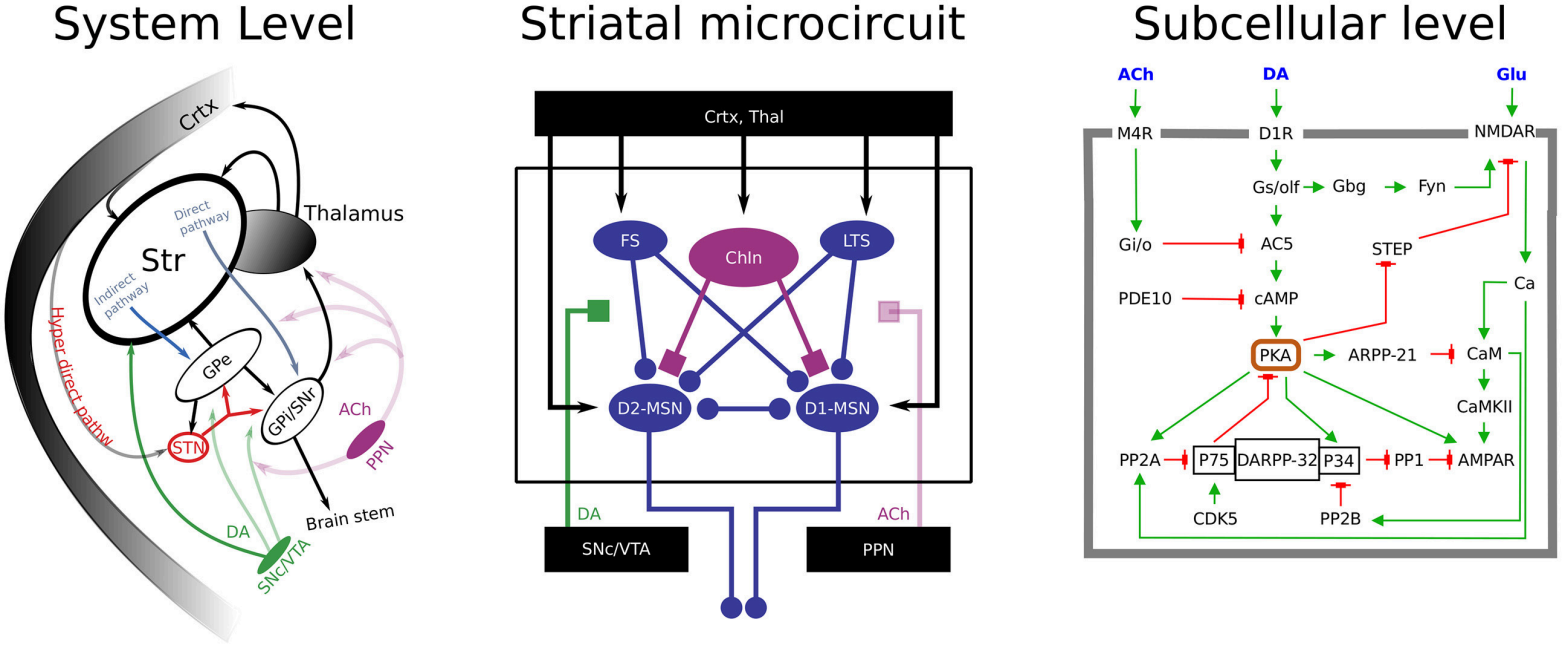
A multi-scale view of basal ganglia. **(Left)** an illustration of the basal ganglia macro-circuitry, showing the glutamatergic and dopaminergic afferent inputs to striatum, as well as the projections via the direct and indirect pathways. Ca 50% of MSNs belong to the direct pathways, they carry dopamine type 1 receptors and project to GPi/SNr (D1-MSN). The other MSNs express dopamine type 2 receptors and project to GPe (globus pallidus externa) before reaching basal ganglia output structures (D2-MSN). At rest these output structure neurons keep motor programs in the brain stem and thalamus under tonic inhibition. When e.g., direct pathway GABAergic MSNs in the striatum become active they inhibit these tonically active neurons, and thereby the inhibition from basal ganglia onto the action/motor centers is relieved. This is the basis for the classical model of basal ganglia function in selection of (motor) actions. **(Middle)** a schematic representation of the striatal micro-circuitry. The main neuron type in the striatum is the medium spiny neuron (MSN), constituting ~95% of striatal neurons. MSNs are the projection neurons from the striatum and receive convergent excitatory glutamatergic input from cortex and thalamus, inhibitory GABAergic input from neighboring MSNs and striatal fast-spiking (FS) and low-threshold spiking (LTS) neurons, cholinergic (ACh) input from cholinergic interneurons (ChINs) and pedunculopontine nucleus (PPN), and dopaminergic input from substantia nigra compacta (SNc). **(Right)** Examples of receptor induces cascades involved in controlling LTP in the cortico-striatal synapse.

As the largest input nucleus of the BG, the *striatum* plays a key role in regulating the information flow through these loops. The principal neuron of striatum is the medium spiny neuron (MSN) that can be further divided into two subpopulations based on DA type 1 (D1R) or DA type 2 (D2R) receptor expression. The two MSN subpopulations are classically thought to have opposing roles in the circuit. The D1R expressing MSN (D1-MSN) is in this model thought to facilitate the initiation of action/behavior via activation of the so called “*direct pathway*” of the BG, and this is enhanced when DA is increased (Gerfen and Surmeier, 2011). MSNs of this type directly inhibits the BG output stages (see Figure 1) which in turn inhibits downstream targets. Activation of these MSNs therefore leads to disinhibition of downstream targets. In addition to the direct pathway, there is the “*indirect pathway,”* starting with the D2R expressing MSN (D2-MSN), which inhibits actions/behaviors (Gerfen and Surmeier, 2011). This “classical” model of the role of the direct- and indirect pathways have in recent years been challenged (Cui et al., 2013; Tecuapetla et al., 2016) but there are also data in support of such functional generalization (Kreitzer and Berke, 2011; Kravitz et al., 2012).

The effects of DA are widespread and affect the system via different receptor dependent mechanisms. Such “small scale effects” can only be understood as a part of the larger system. Because of that, and considering that this study is part of a special edition on neuromodulation, we will briefly highlight the interdependencies between the BG structures and the neuromodulatory systems to provide a wider perspective before we focus on specific aspects of the neuromodulation.

### DA effects over multiple structural and temporal scales

Modulatory effects of DA are found throughout the entire BG system, influencing neuronal excitability and synaptic efficacy across all of its nuclei (see Table 1). It is, however, not well understood to what extent the resulting system level effects are relying mainly on the alteration of membrane excitability and/or the modification of transmitter release and synaptic short-term-plasticity. Also in addition to the acute effects, secondary changes occur, such as synaptic plasticity and/or homeostasis. Such secondary changes play crucial roles in the development, progression and side effects of treatments to diseases, such as Parkinson's disease (PD) and drug addiction.

**Table 1:** Effect of dopamine on neuron excitability and synaptic connectivity in basal ganglia nuclei.

**Nucleus**	**Neuron**	**Effect**	**Source**
Striatum	D1-MSN	Increased excitability	Surmeier et al., 2007; Planert et al., 2013
	D2-MSN	Decreased excitability (variable effect)	Hernández-López et al., 2000; Surmeier et al., 2007; Planert et al., 2013
	FSN	Increased excitability	Bracci et al., 2002; Centonze et al., 2003
	ChIN	Increased excitability (D1-like), hyperpolarization (d2-like)	Aosaki et al., 1998; Centonze et al., 2003; Chuhma et al., 2014
GPe		Diverse effect, mostly increased excitability	Napier et al., 1991; Mamad et al., 2015; Hegeman et al., 2016
GPi		Diverse effect, decreased activity in PD (D1-like)	Ruskin et al., 2002
SNr		Increased excitability	Zhou et al., 2009
SNc	Calb+, Calb–	Membrane hyperpolarization (Calb+/Calb–, D2)/increased rebound spiking (Calb–, D2)	Lacey et al., 1990; Beckstead et al., 2004; Evans et al., 2017
STN		Increased excitability	Mintz et al., 1986; Ni et al., 2001; Zhu et al., 2002; Cragg et al., 2004
**Projection**	**Transmitter**	**Effect**	**Source**
**STRIATAL AFFERENTS**			
Ctx/Th → MSN	Glu	Weakening efficacy	Cepeda et al., 1993; Hernández-Echeagaray et al., 2004
Ctx/Th → FSN	Glu	No effect	Bracci et al., 2002
Th → ChIN	Glu	No effect	Pisani et al., 2000
D1-MSN → MSN	GABA	Strengthening efficacy (D1-like)/No effect (DA)	Harsing and Zigmond, 1997; Guzmán et al., 2003
D2-MSN → MSN	GABA	Weakening efficacy	Harsing and Zigmond, 1997; Guzmán et al., 2003
FSN → MSN	GABA	Weakening efficacy (dStr), diverse effect (NAc)	Kohnomi et al., 2012; Nieto Mendoza and Hernandez Echeagaray, 2015
(unspec) → FSN	GABA	Weakening efficacy	Bracci et al., 2002
Npas1+ GPe → MSN	GABA	Weakening efficacy (DA, chronic)	Glajch et al., 2016
ChIN → MSN	ACh	Weakening efficacy	Pisani et al., 2000; Tritsch and Sabatini, 2012
ChIN → ChIN	ACh	Weakening efficacy (D2-like, presyn)	Pisani et al., 2000
(unspec) → ChIN	GABA	Weakening efficacy (D2-like, presyn)	Pisani et al., 2000
**PALLIDAL AFFERENTS**			
D1-MSN → GPe	GABA	No effect (collaterals)	Chuhma et al., 2011
D2-MSN → GPe	GABA	Weakening efficacy	Cooper and Stanford, 2001; Chuhma et al., 2011
GPe → GPe	GABA	Weakening efficacy	Floran et al., 1997
STN → GPe	Glu	Weakening efficacy (D2-like) or strengthening efficacy (D1-like)	Hernández et al., 2006, 2007
D1-MSN → GPi	GABA	Strengthening efficacy (D1-like, postsyn)	Lavian et al., 2017
GPe → GPi	GABA	Weakening efficacy (D2-like, presyn, prototypical)	Lavian et al., 2017
**NIGRAL AFFERENTS**			
D1-MSN → SNr	GABA	Strengthening efficacy	Yanovsky et al., 2003; Kliem et al., 2007; Chuhma et al., 2011
D1-MSN → SNc	GABA	Strengthening efficacy (D1-like)	Yanovsky et al., 2003
SNr → SNc	GABA	No effect (D1-like)	Yanovsky et al., 2003
**AFFERENTS OF SUBTHALAMIC NUCLEUS**			
Ctx/Th → STN	Glu	Weakening efficacy	Shen and Johnson, 2000
GPe → STN	GABA	Weakening efficacy	Shen and Johnson, 2000; Baufreton and Bevan, 2008

The dopaminergic modulation is relatively more well-studied in the striatum and its principal neurons than in other BG structures. The current view that dopaminergic innervation to MSNs is “excitatory” and “inhibitory” to the direct and indirect pathway, respectively (Gerfen and Surmeier, 2011), would predict that loss of dopamine results in an imbalance between the activities of these two pathways (Albin et al., 1989; Mallet et al., 2006; López-Huerta et al., 2013; Fieblinger et al., 2014).

Recent research highlights that not only an appropriate background DA level is needed for an appropriate functioning of the BG, but transient (phasic; over the order of 100 ms) regulation of the striatal DA levels is needed for ongoing fast-timescale motor control (Howe and Dombeck, 2016). In this case a fast regulation of the membrane excitability might take place via the activation of subcellular signaling cascades affecting various membrane conductances in selected neuronal populations in striatum.

A dynamic DA signal is also thought to drive reward learning by signaling unexpected reward (Schultz et al., 1997; Schultz, 2002). During reward learning long-term synaptic changes are assumed to take place in, for instance, the excitatory cortico-striatal synapses (Centonze et al., 2003; Calabresi et al., 2006; Wang et al., 2006; Wickens, 2009; Surmeier et al., 2011). The intracellular cAMP/PKA signaling (see Figure 1) is important for cortico-striatal LTP (Shen et al., 2008; Kheirbek et al., 2009; Castro et al., 2013). Additionally, dopamine dependent PKA signaling in D1 MSNs also creates an “eligibility trace-like” integration time window for associative learning (Yagishita et al., 2014), possibly via striatally enriched phosphoproteins (Nair et al., 2016).

At the systems level, the extensive research using animal models of PD have been useful to illustrate how the presence or absence of dopamine, affects network activity in behaving animals. In these PD models, death of DAergic neurons in substantia nigra pars compacta (SNc) is induced through application of toxins such as 6-OHDA (in rodents) and MPTP (in non-human primates). This results in major changes in the activity of the BG sub-networks. For instance, both in rodents and non-human primates, DA depletion leads to pathological beta band oscillations (15–30 Hz) of Subthalamic nucleus (STN)-Globus pallidus externa (GPe) populations (Hammond et al., 2007). These oscillations are accompanied by an increase in the firing rate of STN neurons, a corresponding decrease in the firing rate of GPe neurons, and increased synchrony between STN and GPe neurons (Raz et al., 2000; Mallet et al., 2008). Also, the ongoing firing rate of D2-MSNs is significantly increased (Mallet et al., 2006). Furthermore, dopamine depletion increases spike bursting in both STN and GPe (Tachibana et al., 2011; Nambu and Tachibana, 2014), affects the phase locking of GPe neuron activity to cortical slow-wave oscillations (Mallet et al., 2008) and increases correlations between the two principal types of neurons within GPe (Mallet et al., 2008, 2012).

Secondary and compensatory mechanisms come into play if the change in DA drive persists (Cadet et al., 1991; Przedborski et al., 1995; Courtiere et al., 2005; Capper-Loup and Kaelin-Lang, 2013; Golden et al., 2013). Thus, in animal models of DA depletion the acute effects are accompanied or followed by other alterations in the circuitry. In PD patients, in addition to the well-studied motor impairments, these patients also suffer from sensory impairments such as alterations of olfactory, tactile, nociceptive, thermal, and proprioceptive perception (Artieda et al., 1992; Sathian et al., 1997; Boecker et al., 1999; Conte et al., 2013), including impairments in bilateral tactile discrimination (Sathian et al., 1997; Zia et al., 2003). It was recently shown that DA depletion affects both the intrinsic properties of MSNs and sensory processing in the mouse striatum (Ketzef et al., 2017). MSNs in intact mice differ in their input resistance and excitability both *in vivo* and *in vitro*, with D2-MSN having larger values in both parameters (see also Maurice et al., 2015, *in vitro* findings, and Mallet et al., 2006 extracellular *in vivo* data). D1-MSNs normally separate responses to ipsi- and contralateral whisker stimulation better than D2-MSNs (Reig and Silberberg, 2014). When DA is depleted from the striatum, especially D1-MSNs lose this lateral encoding (Ketzef et al., 2017). Also, input resistance and excitability of the D1-MSN is increased following DA depletion, reducing the difference between the two types of MSN observed under control conditions (Maurice et al., 2015; Ketzef et al., 2017; but see also studies in rat Tseng et al., 2001; Mallet et al., 2006). These types of findings point toward compensatory mechanisms playing a role, in such a way that acute blockades of DA receptors can have opposite effects compared to chronic alterations.

### DA-ACh interplay

In addition to DA, ACh is likewise crucial for BG function. It has been assumed since the early 1960s that ACh and DA counterbalance one another, such that an increase in ACh is associated with a decrease in DAergic effects in the striatum (Barbeau, 1962; McGeer et al., 1974). Thus, anti-cholinergic drugs were often prescribed to treat Parkinson's Disease (Duvoisin, 1967). Since then, treatment has focused on increasing dopamine levels through administration of L-DOPA. However, newer work suggests that specific muscarinic receptors (M1, M4) could be a potential target for more specific treatments, reducing negative side-effects (Xiang et al., 2012; Ztaou et al., 2016). Yet another example of the importance of the balance between DA and ACh relates to the use of recreational drugs, such as cocaine and morphine, that lead to elevated DA levels in the ventral striatum (Hikida et al., 2003). As ACh and DA are believed to serve antagonistic roles, increasing cholinergic tone through acetylcholinesterase inhibitors can suppress addictive symptoms (Staley et al., 2006). Maintaining a proper cholinergic tone in the BG then appears crucial in preventing addiction.

The cellular bases of the observed modulatory effects of ACh on the striatal network is starting to be understood (reviewed by Oldenburg and Ding, 2011, etc.). Subcellular effects of the interaction between ACh and DA are also relatively well understood. Interestingly, cholinergic interneurons (ChINs), which normally are tonically active, may show a pause in their activity when DA is transiently elevated during reward learning (Aosaki et al., 1994, 1995, 2010; Morris et al., 2004). The mechanism responsible for the pause is at least partly mediated by activation of D2 receptors on the ChINs, as described by Gerfen and Surmeier (2011). Computational modeling has further shown that such coordinated DA and ACh interaction could set up a time window for the required activation of LTP-dependent subcellular signaling, e.g., PKA, in striatal D1-MSNs (see Figure 1; Nair et al., 2015). On the other hand, in the indirect pathways neurons, a DA dip (as seen during omitted but expected rewards or following aversive stimuli) is hypothesized to gate adenosine dependent PKA signaling, which controls LTP in these neurons (Shen et al., 2008; Yu et al., 2009; Nair et al., 2015; Yapo et al., 2017). These effects of co-occurring ACh/DA (in D1-MSNs) and DA/adenosine-dependent plasticity (in D2-MSNs) could be further modulated by other neuromodulators such as the serotonergic system representing additional aspects of the emotional state of the organism (Tanaka et al., 2007; Seo et al., 2008). As for DA, sub-second ACh burst-pause dynamics might also be important for controlling the membrane excitability during ongoing behavior.

Thus, there is ample experimental evidence that ACh and DA can regulate synaptic plasticity and cellular excitability. This can mechanistically take place through joint regulations of the same receptor activated pathways. Given the importance of an adequate balance and timing between the activation of the different neuromodulators it might not come as a surprise that these same neuro-modulatory systems are also directly influencing and controlling each other. Threlfell et al. (2012) further highlighted these dynamic interdependencies when they reported that synchronous activity of ChINs was capable of driving DA release through local axo-axonal collaterals. Striatal ChINs could thus dictate the local dopaminergic tone, regardless of the activity of the midbrain neurons responsible for these collaterals. Moreover, it was recently shown that putative DA axons co-release GABA and glutamate when activated (Tritsch et al., 2012), suggesting that activation of DA axons mediates various effects by activation of different receptors with different time courses (Straub et al., 2014). Work by Nelson et al. (2014) showed that such GABA co-release can also be initiated by synchronized activity of ChINs, thus forming a negative feedback loop.

More subtle effects have also been observed: ChINs exhibit strong feed-forward inhibitory connections onto neighboring ChINs as shown by Sullivan et al. (2008), presumably mediated by GABAergic neurons. This recurrent lateral inhibition is well suited to synchronize ChINs to a degree necessary to elicit DA release. While the nature of the intermediate neuron is not yet known, it too appears to be modulated by DA, possibly through a D2 mediated inhibition of presynaptic terminals via N-type Calcium Channels, as demonstrated by Momiyama and Nishijo (2017). Taken together, these interactions point toward a spatio-temporal coordination of recurrent control between the cholinergic and dopaminergic systems.

### Control of the neuromodulatory systems through the BG network

Not only do the DA and ACh systems control the BG circuitry and more specifically each other in a dynamic manner, but in similar ways as the BG output nuclei control (motor-)targets in the brainstem (see Figure 1) signaling through the BG directly regulates the activity of midbrain dopaminergic- as well as brain stem cholinergic nuclei. The striatum is divided into a matrisome and striosome compartment (Graybiel, 1984; Crittenden et al., 2016). Whereas the matrisome compartment is involved in the control of motion via the direct and indirect pathways, the striosome compartment is engaged in controlling the level of activity in the DA neurons. The MSNs of the striosomes project directly to the DA neurons and thus provide inhibition, but they also project to a subpopulation of glutamatergic neurons in globus pallidus (GP) that in turn projects to the lateral habenula (LH). The LH projects preferentially to a GABAergic nucleus that in turn inhibits the DA neurons (Stephenson-Jones et al., 2012, 2013, 2016). Aversive stimuli lead to an enhanced activity in habenula-projecting globus pallidus (GPh), and the LH (Matsumoto and Hikosaka, 2007; Hong and Hikosaka, 2008; Stephenson-Jones et al., 2016) and to a net inhibition of the DA activity, whereas reward type stimuli causes a decrease of the GPh/LH and a disinhibition of the DA neurons (i.e., an increase). It appears that the striosomes thus are engaged in regulating the activity level of DA neurons directly and via the GPh/LH link. It hence appears as if the striosomes and related circuitry are engaged in the evaluation of the result of different motor tasks, which clearly is a very important aspect in terms of motor learning, not least in the reinforcement perspective (Stephenson-Jones et al., 2013, 2016; Grillner and Robertson, 2016).

### Simulations as tool to investigate the dynamics of neuromodulation and for integrating data regarding da effects on excitability

Experimental data from the systems-, cellular-, and subcellular levels are all possible to align with e.g., the general views that the dopaminergic innervation can control learning in the BG and the moment to moment balance between the direct- and indirect pathways, but the quantitative dependencies between neuromodulation and changes in the BG function is not well understood, but rather fragments of experimental findings are distributed over many different published studies with little attempts to synthesize the knowledge in a quantitative manner. Hence, to support our understanding and allow formation of testable and more quantitative predictions, models spanning the subcellular—microcircuit—systems level are useful tools.

To create hypotheses how effects of neuromodulation on membrane excitability and synaptic properties are linked to BG systems level function (for instance “action selection”) and/or BG network dynamics, theory- and simulation based research is needed to drive understanding. BG modeling faces the challenge that quantitative constraints on effective connectivity between different BG sub-nuclei is not available and often has to be indirectly estimated (e.g., Bahuguna et al., 2017). Despite this challenge there has been attempts to integrate most of the DA dependent modifications from Table 1 in large-scale computational models of the BG system, see for example (Lindahl and Hellgren Kotaleski, 2016). Besides full models of the Cortico-BG loop, models of striatum (e.g., Humphries et al., 2009; Ponzi and Wickens, 2010; Yim et al., 2011; Tomkins et al., 2013; Bahuguna et al., 2015; Berthet et al., 2016; Belić et al., 2017; Spreizer et al., 2017) and STN-GPe network (e.g., Humphries et al., 2006; Leblois et al., 2006; Kumar et al., 2011; Pavlides et al., 2015; Corbit et al., 2016; Schwab et al., 2017), have been widely used in predicting underlying mechanisms for how various functional or dynamical phenomena described above may arise.

Here we address the question: how the wide spread effects of DA on single channels or receptors can drive the changes in excitability seen in *in vitro* experimentally (typically mapping to “static” effects) and during ongoing behavior *in vivo* (also considering “dynamic” effects). Specifically, we address the question whether the experimentally reported intracellular mechanisms are fast enough to give rise to the observed behavior, which seem to take place in the sub-second time scale (Howe and Dombeck, 2016). Furthermore, we highlight the importance of quantitative measurements of the dynamics in the subcellular signaling. The method we use is multiscale modeling, spanning the cellular-subcellular levels, which gives us access to (and full control over) the state of the cell. As a modeling system we use the D1-MSN of the direct pathway, including an intracellular receptor induced cascade connecting transient DA release to single channel conductances.

## Methods

### Model

In this study a biophysically detailed compartmentalized model is used, which gives us complete control over the state of the cell. In such models, a cell is divided into a finite number of “compartments.” Each compartment is an electrical circuit that is set to represent the electrical properties of a small part of the neuron. A few compartments are for example used to model the behavior of the axon initial segment and the soma while multiple compartments are used for the dendritic tree. The compartments of the model are sequentially connected, providing a representation of the electrical circuit of the whole cell. Current flow over the membrane is modeled using passive, ion concentration dependent, and voltage dependent ion channels, inserted non-uniformly into the compartments. Current flow between compartments are proportional to the voltage difference between neighboring compartments. External currents are given in the form of current injections and synaptic currents. The current injections are modeled as constant currents given to one of the somatic compartments. Synaptic currents are placed in the dendritic compartments and modeled with amplitudes given by time dependent exponential functions. DA modulation of ion channels and synaptic currents (“mechanisms”) are modeled using a fixed or time varying change of mechanism conductance. The time varying modulation is implemented by connecting the mechanism conductance to the concentration of intracellular substrates, dynamically changed when dopamine binds to receptors in the cell membrane. A more detailed description of various aspects of the model is given below.

**Figure 2:**
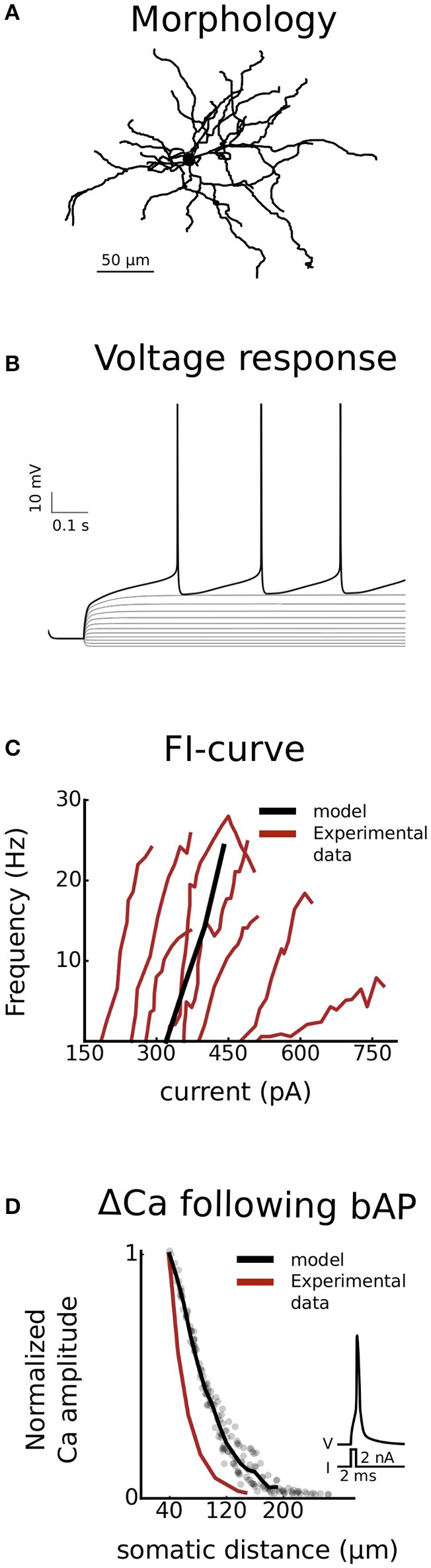
Validation. The model is validated against experimental data from striatonigral medium spiny neurons of the direct basal ganglia pathway. Both somatic (**B** and **C**) as well as dendritic excitability **(D)** is validated. In **(A)**, the dendritic arborisation of the morphology is shown. **(B)** is showing the voltage response of the cell, following current injections ranging from −100 to 340 pA in steps of 40 pA. In **(C)** the current-frequency curve of the model is plotted together with experimental curves from Planert et al. (2013). In **(D)** the dendritic excitability is validated as the local change in calcium concentration as a function of somatic distance following a backpropagating action potential (Day et al., 2008).

The D1-MSN model used in this project is derived from a generic MSN model previously used in our group (Du et al., 2017). The morphology of the model is based on a digitally reconstructed MSN (Figure 2A) and the behavior of the model was tuned to fit experimental data from D1-MSN in the form of rheobase current, current-frequency curve (Planert et al., 2013) and calcium response to backpropagating action potential (Day et al., 2008) (Figures 2B–D). Kinetics of ion channels were increased using Q-factors whenever possible to map experimental behavior in room temperature to physiological temperatures (about 35°C). The setup of the model has been described in detail before (Evans et al., 2012; Paille et al., 2013; Du et al., 2017). Here we will therefore give a general overview with focus on updates introduced in the current version.

The model was built in NEURON+Python (v7.4, Hines and Carnevale, 1997). Simulations were performed on standard laptops with the Linux operating system Ubuntu 16.04 and on resources provided by the Swedish National Infrastructure for Computing (SNIC) at PDC KTH. The model is uploaded to modelDB.

#### Morphology

Morphological reconstruction of the principal cell from dorsolateral striatum of the mouse was used for the MSN model (Cazorla et al., 2012; neuromorpho.org, archive Kellendonk, ID NMO_08390). Spatial and structural data was supplemented with distribution of dendritic diameters from another set of reconstructions (Martone et al., 2003; neuromorpho.org, archive Martone, ID NMO_04518 through NMO_04523) where it was specifically addressed. At any given node, the dendritic diameter was assigned a value, in micrometers,

$$\begin{array}{l} d=max\left(d_{1},\;d_{2}\right),\;d_{1}=0.001\times L+0.87,\;d_{2}=d_{p}\times e^{-l\times 0.08} \end{array}$$

where *L* is the total dendritic length of the branch rooted at the given node, *d*_*p*_ is the diameter of the parent node (in the dendrite or soma for the first node of the trunk), and *l* is the distance to the parent node. Four additional nodes were added to the morphology, two somatic and two axonal. The length and diameter of the soma were 12.2 and 11.2 μm, respectively, while the axonal initial segment had a total length of 60 μm and a diameter of 1 μm. The morphology is shown in Figure 2A.

#### Passive properties

The passive properties of the model were uniformly set to values within accepted range. The specific axial resistance, Ra, was decreased with this in mind from 400 Ohm/cm (Du et al., 2017) to 150 Ohm/cm. A change that brought it into the range of most commonly reported values (70–220) over multiple brain areas (Bekkers and Stevens, 1996; Stuart and Spruston, 1998; Roth and Hausser, 2001; Golding et al., 2005). The specific capacitance Cm, was set to 1 μF/cm^2^. Leak conductance was set to 1.25e-5 S/cm^2^ and reversal potential of leak to −70 mV. Reversal potential of Potassium and Sodium were set to −85 (Podda et al., 2010) and 50 mV (Wolf et al., 2005), respectively.

#### Active properties

The Sodium and Potassium channels of the model came from the last published version of the model (Du et al., 2017) with some minor updates. The inward rectifying Potassium channel (KIR) was updated with smooth kinetics instead of tabulated values. The inactivation time constant of the fast A-type Potassium channel (Kaf) was decreased to consistently simulate physiological temperatures (the Q-factor was applied to both gates instead of just one of them). The kinetics of the transient fast inactivating sodium channel (Naf) was slowed down at depolarized potentials. This smoothed and decreased the amplitude of the afterhyperpolarization (AHP) and decreased the slope of the FI curve. The Q-factor of the Naf channel was also reduced from 2 to 1.8 to further slowdown the spiking frequency while staying within the experimentally reported range (Schwarz, 1986).

The Ca channels and dynamics were updated from the original version of the model (Wolf et al., 2005). The blocker resistant Ca (CaR) channel was updated with the activation curve fitted to m^3^ kinetics (Evans et al., 2013) and upscaled activation time constant, accordingly. The inactivation time constant was implemented using smooth fit to experimental data (Brevi et al., 2001). The long-lasting Ca (CaL) channel was implemented in two versions, CaL1.2 and CaL1.3. The kinetics of these channels followed mh and m^2^h, respectively. The activation time constant of both channels were also refitted to avoid singularities. The neuronal, N-type Ca (CaN) channel followed m^2^h kinetics (Kasai and Neher, 1992), where activation of m^2^ was fitted to experimental data (Bargas et al., 1994; Evans et al., 2012). The activation time constant was scaled up as well (Kasai and Neher, 1992). The transient opening T-type Ca (CaT) channel was also implemented in two versions, CaT3.2 and CaT3.3. Both versions followed m^3^h kinetics (Crunelli et al., 2005; Evans et al., 2013) with upscaled activation kinetics accordingly. The Ca dependent small conductance Potassium (SK) channel was implemented with smooth activation of Ca dependence (Evans et al., 2013).

Uniform channel distribution was used unless experimental data suggested different and/or other distributions gave a better fit to experimental data. The distribution of the Kaf channel in the dendrites was set to increase with increased somatic distance while the slowly inactivating Potassium (Kas) channel and the Naf channels were set using the reversed distribution. These non-uniform distributions were used in agreement with experimental data suggesting that Kaf regulates dendritic excitability while Kas and Naf regulates somatic (Day et al., 2008). A similar increase in dendritc Kaf distribution has also been directly demonstrated in the larger pyramidal cells of hippocampus where direct patching of dendrites is possible (Hoffman et al., 1997). The Kas and the Naf channels were also distributed to the spike initiation zone in the axonal initial segment. The rest of the channels followed the same distribution as the earlier versions of the model. Maximal conductance/permeability and channel distribution of all channels are given in Table 2.

**Table 2:** Channel distribution over cell compartment as a function of somatic distance (x).

**Channel**	**Compartment**	**Function type**	**Maximal value (x)**
Naf	Soma	Uniform	9
	Dendrite	Sigmoidal	0.9 ^*^ [0.1 + 0.9/(1 + exp([x-60]/10))]
	Axon	Step	if (x <30): 9.9 else: 9
Kas	Soma	Uniform	0.012
	Dendrite	Exponential	0.0012 ^*^ [1 + 9^*^exp(−x/5)]
	Axon	Uniform	7^*^10^−3^
Kaf	Soma	Uniform	0.11
	Dendrite	Sigmoidal	0.11 ^*^ [1 +0.5/(1 + exp(−[x−120]/30))]
KIR	Soma/dendrite	Uniform	9^*^10^−4^
Kdr	Soma/dendrite	Uniform	7^*^10^−4^
SK	Soma	–	
	Dendrite	Uniform	2^*^10^−5^
BK	Soma/dendrite	Uniform	10^−4^
CaL1.2	Soma/dendrite	Uniform	10^−5^
CaL1.3	Soma/dendrite	Uniform	10^−6^
CaN	Soma	Uniform	3^*^10^−5^
	Dendrite	–	
CaR	Soma/dendrite	Uniform	10^−4^
CaT3.2	Soma	–	
	Dendrite	Sigmoidal	10^−7^ / (1 + exp(−[x−70]/4.5))
CaT3.3	Soma	–	
	Dendrite	Sigmoidal	10^−8^ / (1 + exp(−[x−70]/4.5))

*Maximal value here stands for permeability (cm/S), for the Ca channels, and conductance (S/cm^2^), for the rest of the channels*.

#### Synaptic noise

Synaptic noise was modeled by placing one randomly activated glutamatergic and GABAergic synapse, respectively, in each section of the cell. Each synapse in the model hence represents many synapses in the real cell. Mean activation frequency and maximal conductance of the noise were tuned to make the cell fluctuate around −70 mV as has been reported *in vivo* (Reig and Silberberg, 2014). The mean activation frequency of glutamatergic synapses was 17 Hz while GABAergic synapses were activated using 4 Hz, giving a ratio of glutamate to GABA of about 4 to 1 (Wilson, 2007). Maximal conductance of the synapses was set to 150 and 450 pS for glutamate and GABA, respectively. NMDA to AMPA ratio of the glutamatergic synapse was set to 1. The synaptic receptors themselves were modeled using double exponential mechanisms with short term depression (STD, Tsodyks et al., 2000). STD were modeled using a time constant of 100 ms while the other time constants of the receptors followed Wolf et al. (2005). The NMDA part of the excitatory synaptic current was also filtered by a voltage dependent magnesium block identical to the one used in Du et al. (2017).

#### Dopamine cascade

Dopamine triggers intracellular signaling events in striatal MSNs, via neuron-type specific DA receptors. This could result in the phosphorylation of various ion channels, thereby affecting the electrical properties of the neuron. Some aspects regarding this have been for the D1-MSNs implemented in NEURON by coupling intracellular signaling with the state variables controlling the electrical properties. The basic building blocks for the intracellular signaling are biochemical reactions, such as binding and enzymatic reactions. Individual reactions were mathematically modeled as differential equations dictated by mass action kinetics. The reactions for DA-dependent intracellular signaling were taken from an existing experimentally-constrained model (Nair et al., 2016). Briefly, the signaling network contains reactions for the activation of D1R by DA which in turn activates the G-protein associated with the receptor. The active G-protein binds to adenylyl cyclase (AC) thereby increasing the enzymatic activity of AC. This increases the cAMP production rate. The network also includes phosphodiesterases responsible for the degradation of cAMP. cAMP activates the main kinase downstream, PKA. PKA could phosphorylate various ion channels. To capture this effect of PKA on its substrate, we included a test substrate which could be efficiently phosphorylated by PKA and dephosphorylated by PP1. The phosphorylation level of this sample substrate is used as a proxy for the extent of PKA-dependent phosphorylation of a given ion channel in the following sections. Even though this approach is simplistic and does not consider channel-specific reaction parameters, it could illustrate the intracellular signaling-triggered transition between various operational states of the neuron.

The dopamine-dependent intracellular signaling was implemented using the standardized markup language, *Systems Biology Markup Language* (SBML) from where it was converted into a NEURON readable mod file. This conversion was done in two steps using NeuroML (Cannon et al., 2014). First the SBML file was exported to the standard NeuroML input format, LEMS, and from that format it was then exported to the mod format. To increase the readability of the mod file, the substrate *ID's* were mapped and exchanged for their respective *names* in the original SBML file. This was done using a custom-made python script. The script read the mod file, line by line, extracting ID's of parameters etc, then searched the SBML file for the corresponding tag and finally exchanged ID for name. To avoid errors due to long variable names the longest names were also reduced to shorter versions using the same script (e.g., rate__revreaction_12 was reduced to r_r_12). The commands used for the conversion were:

1. ./jnml -sbml-import SBML.xlm 1 1
2. ./jnml SBML.xlm_LEMS.xml -neuron
3. python sbml_neuroML_mod_parameterCleaner2.py <mod file from 2> <SBML from 1> <py file from 2>

An application for converting SBML to NEURON, embedding it in a neuron model, and running the code in NEURON from the HBP collaboratory, is currently under construction (https://collab.humanbrainproject.eu). This application uses the NeuroML conversion tool (https://www.neuroml.org/neuron_tools) to create a mechanism.mod file. This mod file can then be inserted within a neuron model. The interface is written as an iPython Jupyter notebook and stimulation and plotting of simulation variables is performed from within the notebook.

#### Dopamine modulation of channels

DA modulation was implemented in the model in two ways. First a static modulation was used to investigate how the contribution of individual modulated channels can lead to increased excitability of the D1-MSN (Flores-Barrera et al., 2011; Planert et al., 2013). Here the maximal conductance of individual channels was multiplied by a modulation factor (MF) that stayed constant throughout the simulation. The range of individual MFs were obtained using a thorough literature study, revising and extending the work done by Moyer et al. (2007). Individual simulations could fall in one of three categories based on excitability compared to control; *Less, Equal*, or *More* excitable. Rheobase current was here used as the readout of excitability. To increase the number of simulations in the Equal group, changes smaller than 10 pA compared to control was categorized as equal excitable. A current step of 10 pA corresponded to about 2 mV change in membrane potential close to the spike threshold. This is also within the interval of glutamate uncaging triggered post synaptic potentials used experimentally (Plotkin et al., 2011). This part of the study was designed to simulate an *in vitro* like situation with no ongoing synaptic drive.

Secondly a dynamic modulation was implemented in which the conductance of individual channels was dynamically coupled to substrates of the cascade. In this setup we used random synaptic activation to drive the cell. It was hence designed to simulate an *in vivo* like situation. This second technique was primarily used to investigate timing aspects of the DA modulation.

#### Random modulation of channels

Since the uncertainty underlying channel modulation is large we assessed excitability changes using large sets of randomly combined MFs, extending outside of the reported range. In these simulations one MF per channel were uniformly drawn from the interval 0-2 (with 1 representing zero modulation) and multiplied onto the conductance of the channel. The range of modulation thus corresponds to ±100%. About 170,000 simulations were run in this way. Additional simulations were run in which individual factors were restricted to the range reported in the literature. Around 8,000 simulations were run in each restricted set.

### Spike extraction

Spikes were extracted from the voltage traces by checking where the latter crossed zero mV. This was done by extracting the index of all positive data point from the potential trace, creating small islands in the membrane potential land scape. Since each island represents one spike, one index per island (the first) were chosen and the time points were extracted from the time trace.

### Analysis of data

The standard, *Pearson correlation coefficient* was used to calculate correlations. Specifically, this was done using the *corr* function of the *Pandas* library in the *Python* programming language. Plotting of correlation was done using *Hinton diagram*, with the magnitude of the correlation visualized as the size of a square. The color (orange or black) of the square reported positive or negative correlation, respectively.

### Experimental data

Experimental data were extracted from published articles using an online extraction tool (Rohatgi, 2016).

## Results

DA modulation affects multiple levels and time scales as summarized in the Introduction. Multiple conductances are also affected at the single D1-MSN level. We therefore started by investigating these effects using a literature review. The review of literature revealed that the Naf, Kas, and CaN/P channels are down-regulated by DA (about 20–40, 15–35, and 20–50, respectively); the KIR and the GABA channels have been reported to be both up- and down-regulated (ranging about −20% to +25% and −20% to +40%, respectively); the Kaf channel is reported not to be modulated in striatum, although there is some indirect evidence in support of modulation, see Discussion; and finally the CaL, AMPA, and NMDA channels are all up regulated (at least 20, 0–40, and 20–60%, respectively). The intrinsic and synaptic single channel effects of DA are summarized in Tables 3 and 4, respectively.

**Table 3:** Summary of the literature study on single channel effect of D1R activation in striatum.

**Chan**	**Measure**	**Effect**	**References**	**Animal**	**Preparation**
Naf	I	−38 ± 5%	Schiffmann et al., 1995	Rat	Culture
Naf	I	−24 ± 2%	Zhang et al., 1998	Rat	Dissociated
Naf	I	−22%	Surmeier et al., 1992	Rat	Dissociated
Kas	I	−20%	Kitai and Surmeier, 1993	Rat	Dissociated
KIR	IV	+	Pacheco-Cano et al., 1996.	Rat	Slice
KIR	I	+25%	Zhao et al., 2016	Mice	Slice
KIR	I	–	Podda et al., 2010	Mice	Slice
CaN/P	I	−50%	Zhang et al., 2002	Rat	Dissociated
CaN/P	I	–	Surmeier et al., 1995.	Rat	Dis./Culture
CaL	I	+	Surmeier et al., 1995.		
CaL	Exc	+20%	Galarraga et al., 1997	Rat	Slice
CaL	Exc	+	Flores-Barrera et al., 2011	Mice	Slice
CaL	PD, #Aps	+20%, +34%	Hernandez-Lopez et al., 1997	Rat	Slice

*Some values given in the Effect column are estimates when there were no exact values given. In the Measure column: I stand for current, IV stands for current-voltage profile, Exc stands for excitability, PD stands for plateau duration and #APs stands for number of action potentials*.

**Table 4:** Summary of the literature study on synaptic effect of D1R activation given as percentage of control.

**Measure**	**Effect (%)**	**References**	**Animal**	**Preparation**
	**NMDA (range 20%** → **60%)**			
I (amp, dur)	+25, +30	Flores-Hernandez et al., 2002	Rat and Mice	Dissociated
I (amp, dur)	+26 ± 7, +5 ± 2	Cepeda et al., 1998.	Rat	Slice
I”	+45.6 ± 19	Tong and Gibb, 2008	Rat	Slice
Vm (amp, dur)	+39 ± 14, +22 ± 7	Levine et al., 1996b	Mice	Slice
Vm (area)	+34 ± 9	Levine et al., 1996	Rat	Slice
	**AMPA (Non-NMDA) (range 0%** → **30%)**			
Vm (area)	+6 ± 5	Levine et al., 1996	Rat	Slice
I	+30	Umemiya and Raymond, 1997	Rat	Slice
I	+11 ± 6	Yan et al., 1999	Rat and mice	Dissociated
I	+21 ± 2.5	Price et al., 1999	Rat	Culture
PHOSP	+	Price et al., 1999	Rat	Culture
PHOSP	+300	Snyder et al., 2000	Mice	Slice
PHOSP	+200	Chao et al., 2002	Rat	Culture
PHOSP	+450	Xue et al., 2016	Rat	In vivo
	**GABA (range** −**20%** → +**40%)**			
I	−20	Flores-Hernandez et al., 2000	Rat	Dis./culture
I	−29.7 ± 3.8	Flores-Hernandez et al., 2002	Rat	Dissociated
I	± and no change	Nieto Mendoza and Hernandez Echeagaray, 2015	Mice	Slice
I^*^ I^**^	+84 ± 34 +44	Janssen et al., 2009	Mice	Slice
I	−14.5 ± 0.7	Hernandez-Echeagaray et al., 2007	Mice	Slice

*I stand for current, amp stands for amplitude, dur stands for duration, Vm stand for membrane potential, and PHOSP stands for phosphorylation*.

I” *indicates current in adult animals (reduced in juvenile)*.

I* *indicates tonic current from low levels in young animals*.

I** *stands for tonic current following application of D1R antagonist in adult animals. Some values are estimated from multiple values or graphs*.

### Down-regulation of the kaf channel is necessary to make the cell more excitable

**Figure 3:**
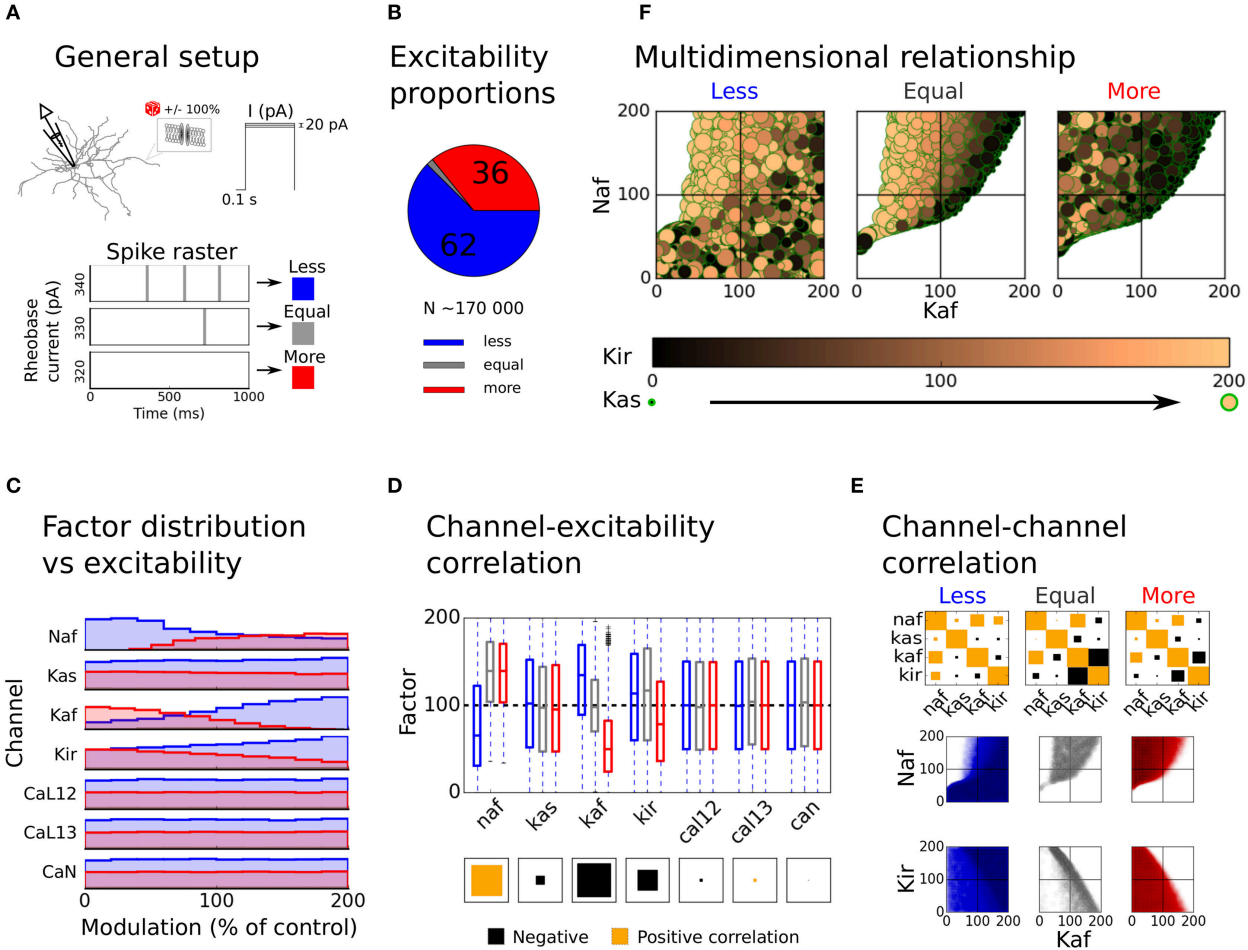
Distribution of modulation factors and overall model excitability. The overall excitability of the uniformly distributed sample set is compared to the unmodulated control case and the contribution of different ion channels to the change in excitability is assessed. In **(A)**, the general setup of the simulation is illustrated. First a random modulation factor (MF) is drawn for each modulated channel and the conductances are scaled accordingly; then the excitability (in the form or rheobase current) of the new setup is compared to the unmodulated control case and is sorted into groups (More, Equal, and Less). A trial that needs at least 10 pA more or less current to spike is sorted into the Less (blue) and More (red) groups, respectively, the rest are categorized as Equal excitable (gray). In **(B)**, the proportions of simulations in each group is shown. In **(C)**, the MF distribution over groups is shown. In **(D)**, the correlation of each channel with excitability is shown as a Hilton diagram. The size of the square is here indicating the magnitude of the correlation while the color of the square shows if the correlation is positive (orange) or negative (black). In **(E)**, the channel-channel correlations as well as pair vice scatter plots are shown for the potassium and sodium channels. In **(F)**, information of all modulated potassium and sodium channels are shown as a function of excitability group. The x and y axes, the color and the size of the dots here shows the modulation factors of the Kaf, Naf, Kir, and Kas channels, respectively.

In this study we specifically investigated how single channel effects could be combined to increase the overall excitability of the D1-MSNs (Flores-Barrera et al., 2011; Planert et al., 2013). To this end we created a large set of simulations where the MF of each ionic conductance was randomly drawn from a sample distribution (See method). This Monte Carlo based approach was used since a systematic variation was not feasible given the possible number of MF combinations. As described in the Methods section, the simulations were then grouped into categories based on their respective rheobase current. All simulations that needed at least 10 pA less or more current to make the cell spike, were grouped into the *More* or *Less* excitable categories, respectively, the rest were categorized as *Equal* excitable (Figure 3A). The proportions of random simulations that fell into each category were then assessed from the full sample set of random simulations. Of all the simulations we considered 36% more excitable, 62% less excitable the rest were considered equal excitable (Figure 3B).

To investigate which channels that had the strongest influence on cell excitability, we next plotted MF distribution as well as pairwise channel-excitability and channel-channel correlations over the three groups (More, Equal, and Less excitable). The distribution of the MFs and the channel-excitability correlations showed that the potassium and sodium channels had the biggest impact on cell excitability, while the Ca channels had little influence (Figures 3C and D). For this reason, the Ca channels were excluded from the rest of the analysis. The channel-channel correlation further showed that the Kaf-Naf channel pair was positively correlated over all three excitability groups, while the Kaf-Kir channel pair was strongly negatively correlated (Equal and More excitable groups, Figure 3E, upper row). The scatter plot of these channel pairs, further showed that while the Less excitable group were generally more uniformly distributed, the Equal and More excitable groups were clearly confined to specific regions. As expected, most simulations with either increase in both the Kir and Kaf channels, or increase in the Kaf and decrease in Naf channel, gave very few More excitable simulations (Figure 3E, lower rows). By adding information from the Kir and Kas channel to the Kaf-Naf scatter plot we could see that More and Equal excitable simulations with relatively large Kaf MFs in general had low MFs of the Kir and Kas channels (Figure 3F). A strong Kaf current can hence be compensated for, to some extent, by a decrease in the other potassium carrying channels (primarily Kir).

**Figure 4:**
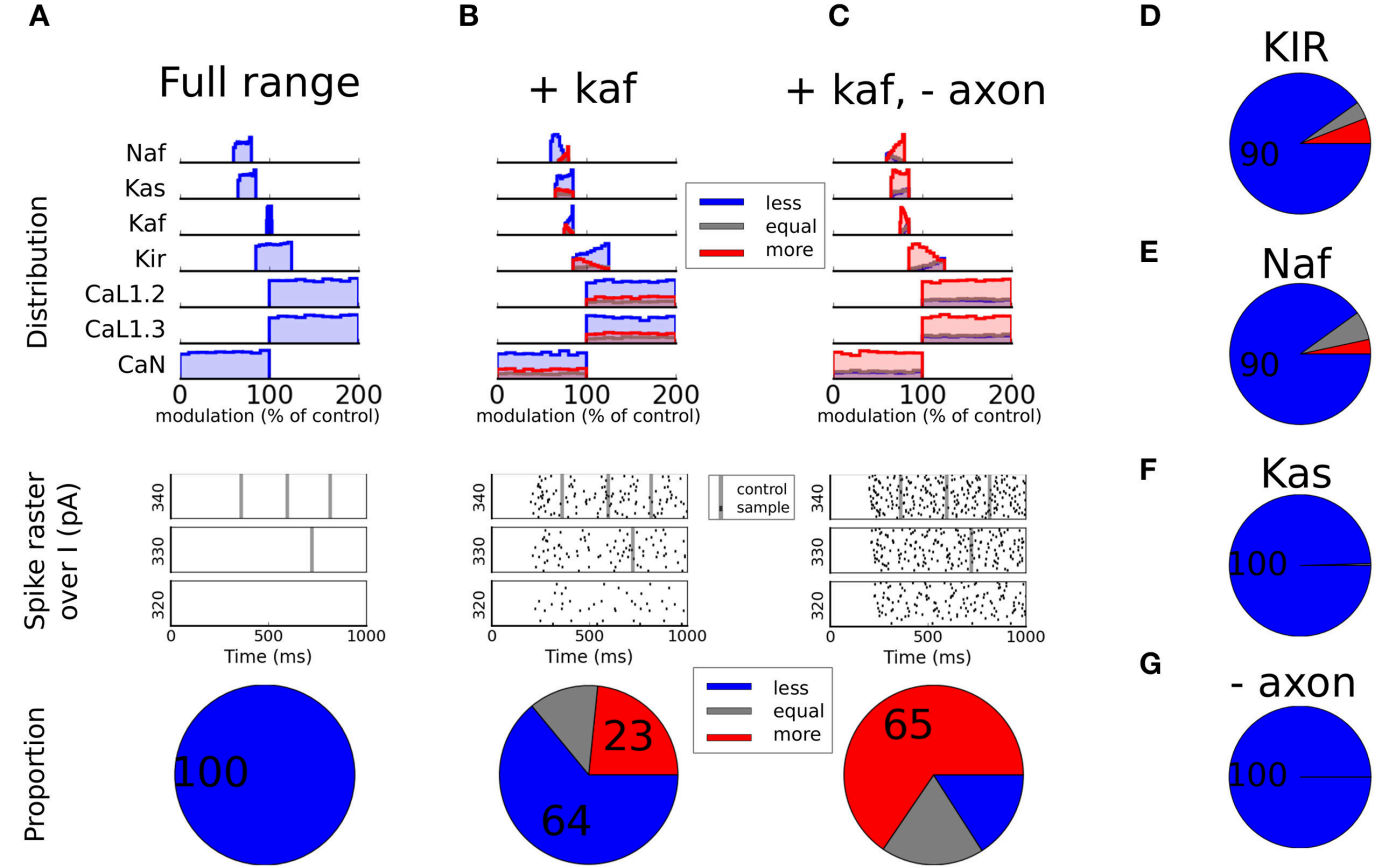
Static modulation. In **(A)**, the modulation factors (MF) are restricted to the experimentally reported range (see Table 3) except for the Ca channels that are kept as free parameters since they did not contribute much to the excitability (see Figures 3C and D). In the top panel the distribution of each channel is shown split over subgroup (L*ess*, E*qual*, or M*ore* excitable). The middle panel shows spike rasters. All black dots here represent spikes from modulated traces while the large gray stripes show the timing of spikes from the unmodulated control case. The bottom panel shows the proportion of the samples of each category. The same type of plots is shown in **(B** and **C)** but here the underlying sample sets have been restricted in different ways. In both plots the Kaf channel is also modulated 80 ± 5% and in **(C)** the axon initial segment is additionally not modulated. In **(D)**, the Kir channel is allowed to reduce more than reported in the literature (down to zero conductance). In **(E)**, the reduction of the fast sodium channel is allowed to reduce less than experimentally reported (up to no modulation). In **(F)**, the slow potassium channel (Kas) is allowed to reduce down to zero conductance. In **(G)**, the same modulation is used as in **(A)**, with the difference that the axon initial segment is not modulated.

When restricting the sample set to only include MFs within the reported range (see Table 3), with no modulation of the Kaf channel, all simulations fell within the Less excitable category (Figure 4A). When also allowing modulation of the Kaf channel (80 ± 5%) it decreased the proportion of Less excitable simulations to 64%. But the proportion of More excitable simulations were still low (23%, Figure 4B). Since the full sample set showed that the Naf and the Kir channels were also influential in increasing the excitability of the cell, we next extended the range of modulation allowed for these channels, one at a time. The upper range of the Naf channel MF extended from 80 to 100% of control (no modulation) while the lower range of the Kir channel MF was decreased from 85 to 0% (zero conductance). Both changes resulted in a reduction in the proportion of Less excitable simulations, but only with a few percent. The proportion of Less excitable simulations was still 90% under both conditions, with only a few percent in the other two groups (Figures 4D and E). Allowing a larger down-regulation of the Kas channel (lower limit from 65 to 0%) also showed only minor effect (Figure 4F). To conclude this section, a down-regulation of the sodium channel by DA in D1-MSN, leads to decreased excitability that to some extent can be compensated for by decreased potassium currents. Primarily the Kaf and the Kir channels are influential in this compensation.

### Non-uniform modulation over cellular compartments

The Naf current in our model is a compound current which is carried by several different members of the sodium channel family. These channels are preferentially distributed to different compartments of the cell. The members that are located in the soma and proximal dendrites are more easily phosphorylated by PKA than the ones in the axon initial segment (Maurice et al., 2001; Hu et al., 2009). Hence, it is possible that different compartments are differently modulated. To test this hypothesis, we performed additional simulations in which the axon initial segment was not modulated by DA, i.e., no channels in the axonal compartments (Naf and Kas) were modulated by DA. To our surprise this did not increase the proportion of simulations that were more excitable following DA modulation (100% were still Less excitable, Figure 4G). If this effect was combined with a decreased Kaf channel, on the other hand, we saw a large increase in excitability (65% were More excitable and only about 15% were Less excitable, Figure 4C).

To conclude this section, if the spike initiation zone in the axon initial segment is not modulated and the Kaf channel is modulated, a large number of MF combinations, within reported range, will make the cell more excitable.

### Is the effect of kaf channel modulation in line with experimental data?

**Figure 5:**
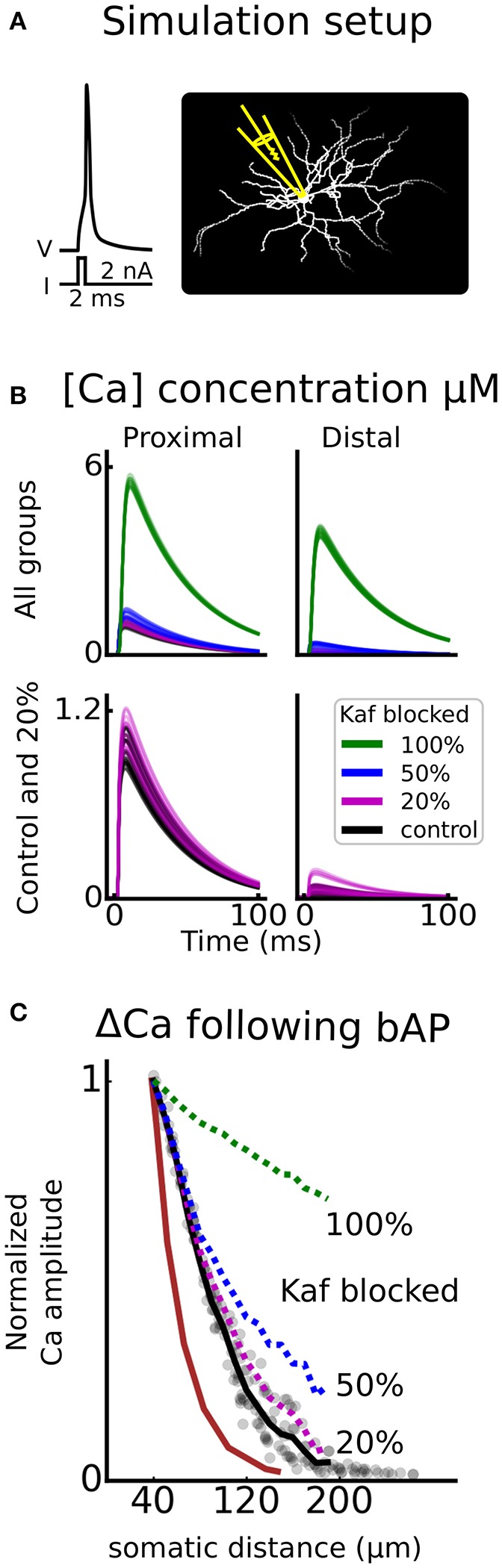
Kaf channel control of calcium influx following a backpropagating action potential. In **(A)**, an illustration of the simulation setup is shown. An action potential is triggered by giving a strong depolarizing current pulse (2 nA, 2 ms) to the soma, and the calcium (Ca) concentration is monitored as a function of somatic distance. Similar to florescent imaging. This setup is repeated for different proportions of Kaf channel blocking; 100, 50, 20, and 0% (control). In **(B)**, example traces of concentration are shown for either proximal (30–50 μm), or distal (170–200 μm) dendritic compartments. The upper panel shows all groups of blocking proportions and the lower panel only holds control and the assumed proportion blocked by dopamine (20%). In **(C)**, the mean change in amplitude of the Ca concentration, is plotted, as a function of somatic distance. The curves are normalized to the proximal location, mimicking experimental data (brown trace, Day et al., 2008).

Since the Kaf channel primarily regulates the dendritic excitability (Day et al., 2008), it is perhaps not that surprising that experimental studies using dissociated cells, fail to find DAergic effects on this channel (Kitai and Surmeier, 1993; Dong and White, 2003). More surprisingly, direct blocking of the Kaf channel, but not local dendritic application of DA, increased the fluorescent signal following a bAP (Day et al., 2008). It was however proposed by the authors that the most likely explanation to their negative finding was that this modulation is not effectively assayed by a single bAP. We therefore asked if DA modulation of the Kaf channel can induce changed excitability while not affecting the bAP? To test this, we repeated the simulation of the bAP, while blocking the Kaf channel partially (to mimic the DA effect) and fully (to mimic the blockage by 4-AP), and compared with the results in Day et al. (2008). The result of this simulation showed that a full block of the channel gave a substantially increased signal while a partial block, in the range of the assumed DA induced modulation (20%), hardly gave any increase at all (Figure 5). Our simulations thus confirms the hypothesis in Day et al. (2008), and strengthen our prediction that increased excitability of D1-MSNs, following dopamine administration, is Kaf channel dependent.

### Excitability in an *in vivo* like situation

In the previous sections we used an *in vitro* like setup where there was no ongoing synaptic activity. Next we asked how modulation of channels in the reported range would affect an *in vivo* like situation where synaptic input would drive the cell. Using the simulation design described in the Methods section, with channels and receptors dynamically modulated by PKA from an intracellular cascade, we created a large set of simulations using random MFs. The range for the factors of different channels were restricted to the ones tested statically in the *in vitro* like situation. The ranges for the AMPA, NMDA and GABA receptors were set to the reported ranges, summarized in Table 4. Since the model was tuned to fluctuate around −70 mV in control condition, it did not spike. For this reason we had only two groups in the analysis, spiking and non-spiking. If the cell spiked within 1 s from the onset of the DA cascade it was categorized as spiking and if it did not, it was categorized as non-spiking. The simulations were further divided into different modulation paradigms. In the first paradigm only AMPA and NMDA channels were modulated (Excitatory), the second also included GABA modulation (Synaptic), the third held only non-receptor gated ion channels (Intrinsic), the fourth held intrinsic and excitatory (No GABA) and in the final all channels were modulated (PKA).

**Figure 6:**
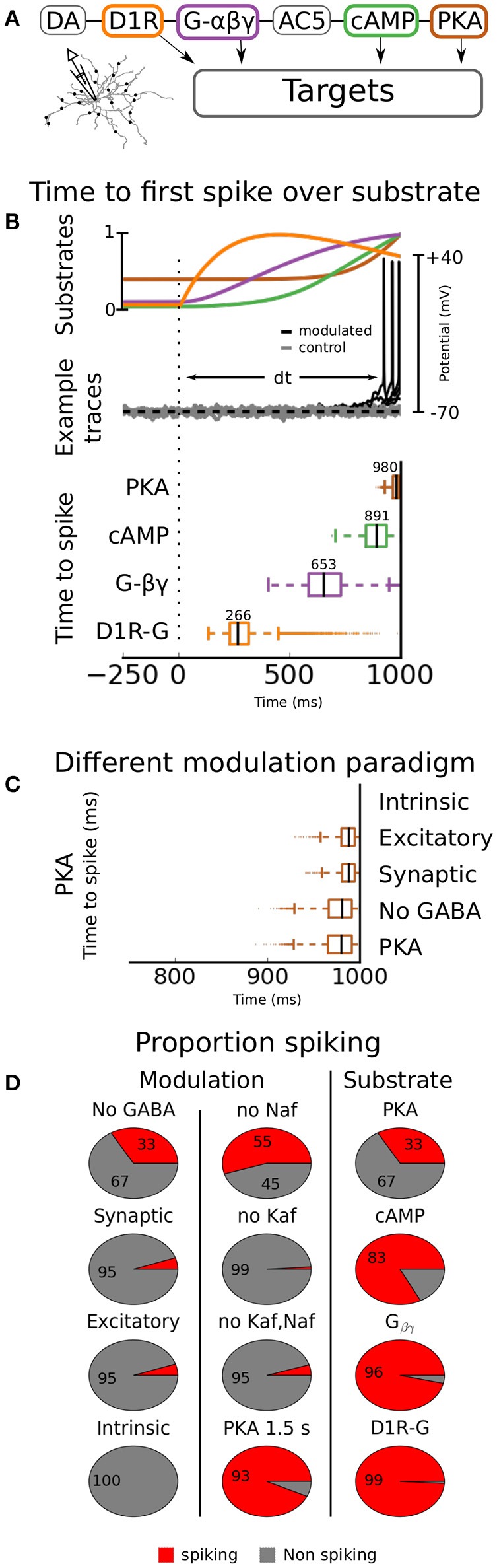
Dynamic modulation. The timing aspects of the DA modulation is assessed by connecting the channel conductances to a DA transient. The transient follows an alpha function with 0.5 μM amplitude and a time constant of 500 ms. In **(A)**, the simulation setup is outlined, showing a simplified illustration of the intracellular cascade. The model is here driven using synaptic activation as in an *in vivo* like situation. In **(B)**, the time to first spike is quantified as a function of modulating substrate (bottom panel). The median value for each substrate is given above respective box. In the top panel the underlying kinetics of the modulating substrates are shown. In the middle panel a few examples of voltage traces are shown. Both modulated (black) and unmodulated control traces (gray) are shown. In **(C)**, different modulation paradigms are used; *Intrinsic* means that only intrinsic ion channels are modulated, *Excitatory* means that only AMPA and NMDA channels are modulated (for range see Table 4), *Synaptic* means that the GABA channels as well as the AMPA and NMDA channels are modulated, *No GABA* means that all channels but the GABA channels are modulated, and in the final group (*PKA*) all channels are modulated. In **(D)** the proportion of traces that spikes are shown as a function of modulating substrate and modulation paradigm (all connected to PKA). Additionally to the ones shown in **(C)**, there are also the groups *No Naf*, *No Kaf*, “*No Kaf, Naf,”* and *PKA 1.5 s*. In the first three groups the simulation is run without modulation of the respective channel(s) mentioned in the title. In the last group the simulation time is extended from 1 to 1.5 s.

The results showed that the excitatory or the intrinsic groups alone triggered spiking in 5 and 0% of the cases, respectively. Combining the two on the other hand, increased the spiking proportion to 33% (No GABA, Figure 6D). If the sodium channels are not modulated in the subsecond time scale the proportion spiking is further increased to 55% (*no Naf*, Figure 6D). Modulating GABA as well had little or no effect since the spiking proportion stayed constant (compare excitatory with synaptic and PKA with the *no GABA* group in Figure 6D). We further saw that the synergy of combining synaptic and intrinsic modulation was gone if the Kaf channel was not modulated (*no Kaf*, Figure 6D). The proportion of spiking simulations was increased when longer duration of the modulation was allowed (up to 1,500 ms, see *PKA 1.5 s*, Figure 6D).

Hence, our simulations show that the combined effects of DA modulation on excitatory synaptic channels and intrinsic ion channels are needed to cause spiking in D1-MSN. We further predict that the mechanism causing the synergy depends on down-regulation of the Kaf channel.

### Kinetic aspects of modulation *in vivo*

Next, we tested how activation of DAergic terminals within striatum can trigger movement within a few hundred milliseconds (Howe and Dombeck, 2016) when channel effects are usually measured over minutes (Planert et al., 2013). For this reason, we coupled various substrates from the intracellular cascade previously mentioned (see Methods), to the channels (see Figure 6A for an illustration), and quantified the time to first spike for each of them (Figure 6B, lower panel, and Figure 6C). For a detailed description on how the coupling was done see Methods. In short, the substrate concentration was set to influence the maximal conductance of the modulated channels. Three of the substrates tested; PKA, cAMP, and Gβγ, have all been proposed as mechanisms of DA modulation for at least some channel (see Table 5). The last one, direct D1R-channel interaction was further tested, despite low credibility, since it would speed up modulation. The reason why we at all considered this is that D1Rs have been shown to co-localize with CaN channels in the membrane of cortical pyramidal neurons (Kisilevsky et al., 2008). However, it is important to note that DA modulation in this study was still G-protein dependent and that the authors failed to find such an organization in striatum.

**Table 5:** Summary of studies reporting mechanism of DA modulation.

**Mechanism**	**Ion/Chan**.	**Reference(s)**
PKA	K^+^	Hoffman and Johnston, 1998; Schrader et al., 2002; Dong and White, 2003; Yang et al., 2013; Zhao et al., 2016
	Na^+^	Schiffmann et al., 1995, 1998; Zhang et al., 1998; Maurice et al., 2001
	Ca^2+^	Surmeier et al., 1995; Zhang et al., 2002.
	AMPA	Price et al., 1999; Yan et al., 1999; Banke et al., 2000; Snyder et al., 2000; Chao et al., 2002; Glovaci et al., 2014
	NMDA	Flores-Hernandez et al., 2002
	GABA	Flores-Hernandez et al., 2000; Nieto Mendoza and Hernandez Echeagaray, 2015
cAMP/PKA	Kir, CaL	Pacheco-Cano et al., 1996.; Hernandez-Lopez et al., 1997; Dong et al., 2004
cAMP	Kir	Podda et al., 2010
G_β_γ	Ca^2+^	Herlitze et al., 1996
D1R-chan interaction	CaN	Kisilevsky et al., 2008
Direct blocking	NMDA	Castro et al., 1999; Cui et al., 2006
tyrosine dep. Pathway	NMDA	Dunah et al., 2004; Hallett et al., 2006

*PKA stands for protein kinase A, cAMP stands for cyclic adenosine monophosphate*.

In this simulation we used modulation of all channels since we showed earlier that this gave the highest proportion of spiking and our main focus in this study was to investigate kinetics of the modulation. We still quantified the proportions of spiking simulations since the different non-linear kinetics of ion channels and substrates might cause unforeseen dynamical effects.

The median first spike resulting from the assumed PKA stimulation was triggered close to the upper limit of 1,000 ms used in the simulation. This delay makes it much too slow to cause the observed behavior. cAMP was slightly faster, about 890 ms. Direct Gβγ interaction decreased the time to first spike to a median of 650 ms while channel-D1R complexes triggered spiking in about 270 ms.

To conclude this section, PKA can in our model trigger spiking with a latency of about 1 s from the onset of DA activation. Other signaling substrates in the intracellular network, closer to the upstream receptor, can trigger spiking faster, but only direct D1R-channel complexes are, in our model, fast enough to explain action initiation within a few hundred milliseconds. Since such connections have not been found within striatum, either the model does not faithfully reproduce the underlying physiological processes (see section Discussion), or modulation of channels and receptors is not the primary mechanism of fast DAergic action initiation. Either way these results highlight that we need to learn more about the kinetics and spatial organization of substrates within neurons under neuromodulation to explain subsecond neuromodulatory effects (see Chuhma et al., 2017).

## Discussion

In this study we briefly reviewed the role of DA in the BG. In an attempt to investigate more specifically how DA brings about major changes in the sub-nuclei of the BG, we here focused on the DAergic effect on D1-MSNs. The choice of D1-MSNs was motivated by the facts that (1) the D1-MSNs are the starting point of the so called direct-pathways (Gerfen, 2004), which is important in action selection (Bahuguna et al., 2015; Lindahl and Hellgren Kotaleski, 2016) (2) D1-MSNs have been extensively studied so much data have been reported that can be incorporated into the model.

We have further shown how computational techniques can be used to integrate the result from different studies to make predictions where experiments are indecisive. Here we exemplified this by investigating specific aspects of DA modulation on the striatal D1-MSN, but similar studies could and perhaps should be conducted for all cell types in the BG. As a starting point for such studies we provide an overview table showing the widespread effect of dopamine modulation in the BG (Table 1). New techniques in the transcriptomics and imaging fields should further be used to provide additional information on the subcellular substrates involved in the modulation over cell types and nuclei, e.g., fluorescent *in situ* RNA sequencing (Lee et al., 2014).

### Modulation of single channels

In this study we found that down-regulation of the Kaf channel was necessary to increase the excitability of the D1-MSN. At first, this result seems to contradict experimental studies, unable to find effects of DA on the fast potassium current (Kitai and Surmeier, 1993; Dong and White, 2003; Day et al., 2008). The first two studies however, are conducted in dissociated cells where most of the dendrites are lost, and the Kaf channel, on the other hand, is most influential in the dendrites (Day et al., 2008). In the third study, Day et al. (2008), further detected no significant change in bAP amplitude following puffing of DA in the dendrites, while blocking of the Kaf channel had an effect. They however hypothesized that the experimental setup was not suited to catch such a change. This is also in accordance with our simulations showing that a small decrease (20%) of Kaf conductance can increase excitability while the bAP amplitude is not much affected (see Figure 5C).

The Kaf channel in the model is a composite channel primarily carried by the Kv4.2 channel (Tkatch et al., 2000) and there is indirect evidence in support of modulation of the Kv4.2 channel. If the channel has formed a complex with an ancillary subunit, the *potassium Channel Interacting Protein 3* (KChiP3), it can be down regulated by PKA (Schrader et al., 2002) and KChiP3 is expressed in striatum (Xiong et al., 2004).

The Kv4.2 channel can further bind to *A-kinase anchoring proteins* (AKAPs) known to enable fast reliable PKA signaling (Lin et al., 2010). Disruption of this anchoring mechanism decreases excitability of pyramidal cells in hippocampus (Lin et al., 2011), providing a link between PKA phosphorylation of the Kv4.2 channel and excitability. Another possibility is that the lack of direct evidence in support of DA modulation of the channel indicates a high degree of phosphorylation under control conditions. This would be consistent with the decreased excitability following disruption of the anchoring mechanism. In such a scenario we would expect to find that other neuromodulators, acting to decrease PKA signaling, would increase the Kaf current and thereby decrease the excitability of the cell.

Phosphorylation, e.g., by PKA has also been shown to decrease the Kaf current in hippocampal pyramidal neurons, by shifting the voltage dependence of the activation gate into more depolarized potentials (Hoffman and Johnston, 1998).

Regarding the Naf channel, DA modulation causes an enhancement of the slow inactivation (SI) mechanism and thereby a decreased current (Carr et al., 2003; Chen et al., 2006). SI is driven by the collapse of the channel pore (Chen et al., 2006; Payandeh et al., 2012), and develops over seconds (Rudy, 1978). The process is hence too slow to be involved in the observed behavior (Howe and Dombeck, 2016) but will likely affect excitability in slice experiments. The experimental setup will further affect the degree of modulation. The SI is not only voltage dependent (Cantrell et al., 1999) but is also affected by other post-translational modifications, such as PKC phosphorylation and methylation level, both of which also modulates sodium currents (Chen et al., 2006; Baek et al., 2014). Given the complexity of small scale effects, it is therefore clear that DA modulation cannot be fully understood alone, but only as a part of the bigger puzzle.

DA modulation of Ca channels had a low influence on excitability in our model. This indicates that either is the base expression level higher than in the model or these channels have primarily other functions. Many intracellular cascades are regulated by Ca, so it is possible that these channels are mostly involved in plasticity and learning. For example, L-type channels are not necessary for LTP induction in striatal slice preparations (Calabresi et al., 2000), or dopamine induced enhancement of NMDA currents in dissociated MSNs (Flores-Hernandez et al., 2002), but is needed for LTD (Paille et al., 2013). The NMDA channel is a prominent source of Ca itself, so increasing this current, due to DA modulation, might facilitate LTP. Some of the Ca channels are also directly coupled to the Ca-dependent potassium channels, and the blocking of these channels have been shown to affect excitability (Hopf et al., 2010).

### Dopaminergic signaling

We here found that if the axon initial segment was not modulated it gave a largely increased proportion of factor combinations that made the cell more excitable. Experimentally there are at least two pieces of evidence in support of such an organization. Firstly, as mentioned in the Result section, some channels that are preferentially distributed to the axon initial segment are less easily phosphorylated by DA (Maurice et al., 2001; Chen et al., 2008; Hu et al., 2009). And secondly the DAergic connection to MSNs is primarily dendritic (Pickel et al., 1981; Freund et al., 1984).

There are also questions regarding the kinetics of the modulation. In our simulations, the only substrate that could trigger a subsecond behavior was direct receptor-channel coupling. Such interaction has not been reported in striatum. To the contrary, the study reporting the connection in prefrontal cortex, failed to find such connection in striatum (Kisilevsky et al., 2008). This leaves a few possible explanations, (1) that the cascade we used is too slow, despite being speeded up to explain the timing dependency in reward learning within striatum (Nair et al., 2016), (2) the channels are more sensitive to substrate change than modeled, (3) the “resting state” of the membrane potential *in vivo* is closer to the spike threshold than what is used in the model, the cell will thereby respond faster to increased excitability, and/or (4) the activation of DAergic terminals activates fast, excitatory ionotropic receptors to a higher degree than previously reported in dorsal striatum (see below and review by Chuhma et al., 2017).

### Compartmentalized organization of intracellular substrates

Specific anchoring proteins, e.g., AKAPs, form large signaling complexes by direct (Gao et al., 1997; Westphal et al., 1999), as well as indirect (Colledge et al., 2000) binding to channels. Disruption of these links cause abolished modulation by DA (Rosenmund et al., 1994; Few et al., 2007) as well as impaired learning (Carlisle et al., 2008; Nithianantharajah et al., 2012). In addition to such functional compartmentalization, neuronal geometry could also play a role into how signals are integrated by intracellular molecular mechanisms (Neves et al., 2008). The signaling model that is currently used, does not take the functional compartmentalization and geometrical aspects explicitly. Rather, the reaction parameters have been selected to capture the overall characteristics of the downstream responses as observed in measurements (Nair et al., 2016).

### Co-release

Co-release of several neurotransmitters from the same presynaptic neurons has been shown in various synaptic pathways in the brain, often involving combinations of “fast” neurotransmitters with slower “neuromodulators” (Yu et al., 2015; also see review by Tritsch et al., 2016). In the striatum, dopaminergic terminals originating from the midbrain (VTA and SNc) were shown to co-release glutamate onto MSNs in the nucleus accumbens (Chuhma et al., 2004; Tecuapetla et al., 2010). Recently, it was shown that dopaminergic terminals in striatum also co-release GABA (Tritsch et al., 2012). While there are still many open questions regarding co-release, e.g., the topographical arrangement, the target selectivity, dynamics, and reuptake mechanisms, it is clear that both types of MSNs and ChINs receive GABA input from dopaminergic neurons as studied in DAT-Cre mice. In a recent study, marked differences in the co-release of dopamine with GABA or glutamate were shown between the nucleus accumbens core and shell regions and dorsal striatum (Chuhma et al., 2014). While inhibitory responses were seen in the dorsal striatum, neurons in the medial shell region of nucleus accumbens responded with ePSPs mediated by glutamate (Chuhma et al., 2014). The strength of ePSPs depended on the postsynaptic striatal cell types, with ChINs responding stronger than MSNs and FS interneurons. These finding demonstrate diverse effects of the midbrain dopamine system onto different striatal regions and neuron types, and further suggest complex interactions between the dopaminergic and cholinergic systems in the striatum.

## Future perspective

Based on the predictions of the model that the fast potassium channel must be modulated by DA to explain the increased excitability of D1-MSNs, we propose that staining is used to elucidate if Kv4.2-KChIP3 complexes are indeed formed within striatum. Another member of the KChIP family (KChIP1) shows a high specificity and can be used as a marker for the striosomal compartment in striatum (Mikula et al., 2009). This indicates that potassium channels of the striosome and matrix compartments are differently modulated by neuromodulators, such as DA. It would therefore be interesting to see if other Kv4-KChIP complexes are formed, and if so how this affects channel currents and modulation. In addition, our results when it comes to subcellular signaling suggest that co-transmission of glutamate, or some other fast, non PKA-dependent mechanism is triggered by activation of DAergic terminals in dorsal striatum. Further research on the intracellular cascades triggered by dopamine, as well as network effects following activation of DAergic terminals is needed in order to elucidate this mechanism.

## Author contributions

All authors contributed actively in collecting information regarding dopamine effects. AN contributed the subcellular cascade model. DK and RL exported the cascade into Neuron and prepared the setup at the HBP Collaboratory. RL, AKK, KD constructed the MSN model. RL performed the simulations and analyzed the data. RL and JH planned the study. All authors contributed to the writing.

### Conflict of interest statement

The handling Editor declared a shared affiliation, though no other collaboration, with one of the authors DK. The authors declare that the research was conducted in the absence of any commercial or financial relationships that could be construed as a potential conflict of interest.
